# Acknowledgment of Reviewers of *NeuroSci* in 2021

**DOI:** 10.3390/neurosci3010006

**Published:** 2022-01-28

**Authors:** NeuroSci Editorial Office

**Affiliations:** MDPI AG, St. Alban-Anlage 66, 4052 Basel, Switzerland

Rigorous peer review is the basis of high-quality academic publishing. Thanks to the efforts of our reviewers, *NeuroSci* was able to maintain its high standards in terms of the quality of its published papers. In addition, in 2021, a median time to first decision of 16 days and a median time to publication of 36 days were achieved. The editors would like to extend their gratitude and recognition to the following reviewers for their precious time and dedication, regardless of whether the papers they reviewed were finally published:
Alatorre-Cruz, Graciela CatalinaLuís, Ana LúciaAlexander, Francis Borgio J.Mahmoudi, ElhamAngireddy, RajeshMartinez, SalvadorAshraf, Ghulam MdMathieu, Di MiceliBadawy, RadwaMejías, RebecaBergeron, DavidMladenović, JelenaBernhardt, NadineMolina-Mula, JesúsBindu, Dhanesh SivadasanMontemurro, NicolaBonsi, PaolaMulherkar, ShalakaBrito, Maria AlexandraMurphy, ElliotBrown, CulumNaumann, MarkusCacialli, PietroPal, AjayCaldieraro, Marco AntonioPennington, CatherineCialfi, DanielaPetráš, MarekCiuman, RaphaelPiotr Michalak, KrzysztofCooke, Bradley M.Pochet, RolandCosta, JuliaPurcell, JeremyEmrani, SheinaRacz, AttilaFeito, JorgeRandazzo, Melissa A.Forrester, GillianRao, AparnaFriedman, RobertRenner, BertoldFusar-Poli, LauraRoy, BidishaGonzalez, BárbaraSafron, AdamGonzalez-Escamilla, GabrielScaini, SimonaGulyaeva, Natalia V.Schackert, GabrieleGupta, ShwetaShah, ShamikHmeadi, OmarShioda, SeijiHung, Yu-ChiangSingh, KavitaIbanez-Molina, Antonio J.Smith, AshleyIyer, Nisha R.Sobaniec, PiotrJembrek, Maja JazvinšćakTorres-Fernández, OrlandoKovac, AndrejVerde, FedericoKrug, Hollis E.Versnel, HuibKuba, Michael J.Villa, ChiaraKumar, ShaileshWaterhouse, LynnLapka, MarekYip, Ping K.Little, R. DanZanon, Renata Graciele


